# Evidence based post graduate training. A systematic review of reviews based on the WFME quality framework

**DOI:** 10.1186/1472-6920-11-80

**Published:** 2011-10-06

**Authors:** Annelies Damen, Roy Remmen, Johan Wens, Dominique Paulus

**Affiliations:** 1 Department of Family Medicine, Centre for General Practice, University of Antwerp, Universiteitsplein 1, 2610 Wilrijk, Belgium; 2 Federaal Kenniscentrum voor de Gezondheidszorg - Centre fédéral d'expertise des soins de santé - Belgian Health Care Knowledge Centre, Kruidtuinlaan 55, 1000 Brussel, Belgium

## Abstract

**Background:**

A framework for high quality in post graduate training has been defined by the World Federation of Medical Education (WFME). The objective of this paper is to perform a systematic review of reviews to find current evidence regarding aspects of quality of post graduate training and to organise the results following the 9 areas of the WFME framework.

**Methods:**

The systematic literature review was conducted in 2009 in Medline Ovid, EMBASE, ERIC and RDRB databases from 1995 onward. The reviews were selected by two independent researchers and a quality appraisal was based on the SIGN tool.

**Results:**

31 reviews met inclusion criteria. The majority of the reviews provided information about the training process (WFME area 2), the assessment of trainees (WFME area 3) and the trainees (WFME area 4). One review covered the area 8 'governance and administration'. No review was found in relation to the mission and outcomes, the evaluation of the training process and the continuous renewal (respectively areas 1, 7 and 9 of the WFME framework).

**Conclusions:**

The majority of the reviews provided information about the training process, the assessment of trainees and the trainees. Indicators used for quality assessment purposes of post graduate training should be based on this evidence but further research is needed for some areas in particular to assess the quality of the training process.

## Background

The debate on quality improvement in post graduate medical education (PME) is ongoing in many countries [1]. In the UK, the Post graduate Medical Education and Training Board (PMETB) developed generic quality standards of training in September 2009 [2]. In the U.S., the Accreditation Council for Graduate Medical Education (ACGME) is a private professional organisation and nowadays responsible for the accreditation of more than 8500 residency and fellowship programs [3]. Other countries, for instance the Netherlands and Canada also developed quality frameworks in PME [4,5].

An international framework for quality of PME has been proposed by the World Federation of Medical Education (WFME, a non-governmental organization related to the World Health Organization). Global Standards for the undergraduate, post graduate and continuing medical education were developed by three international task forces with a broad representation of experts in medical education from all six WHO/WFME Regions [6]. One of these frameworks aims to introduce a generic and comprehensive approach to quality of PME, providing internationally accepted standards and national or even regional recognition of programs [6].

In the framework above many quality indicators and rankings are used for assessing the quality of PME. However they are based on expert consensus and a further interesting step is to know to what extent they are supported by the evidence: the concrete question is to know if the quality found for each area has an impact on the training outcomes e.g. physicians' competencies and ultimately, quality of care [7]. To date, only few studies comprehensively explored the literature of the quality of post graduate medical education [8].

**Figure 1:**
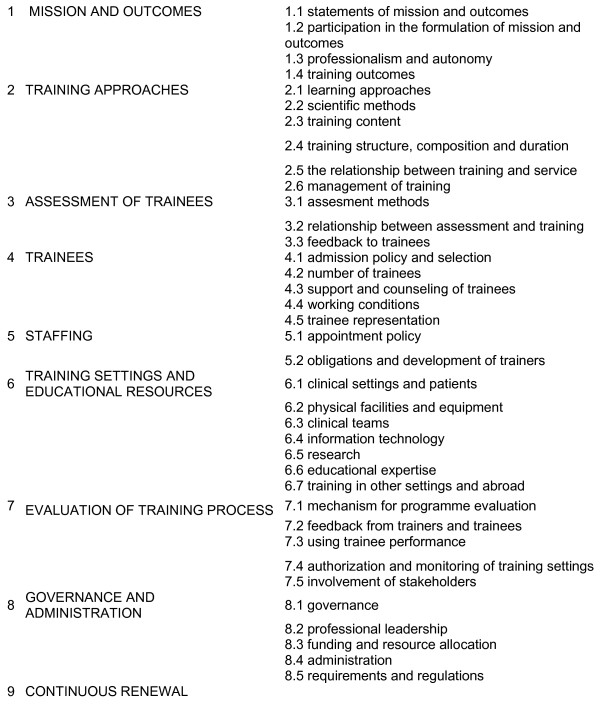
**WFME areas and sub-areas**.

A mapping of the best evidence underlying the WFME framework is an ambitious work. In general, individual studies are biased by local factors and this limits generalising the findings. Therefore systematic reviews have an important role in summarizing and synthesizing evidence in medical education on a wider scale [9]. The objective of this review is to identify, appraise the quality and synthesize the best systematic reviews on post graduate medical education using the WFME standards as a blueprint (Figure 1).

## Methods

### Databases

The systematic literature search was performed from 1995 onwards, using the following data sources: Medline Ovid (April 23, 2009), Embase (Excerpta Medica Database until June 9, 2009), ERIC (Education Recources Information Center) database until July 30, 2009) and RDRB (Research and Development Resource Base database until August 1, 2009) [10,11]. The review included publications in English, French and Dutch.

A complementary manual search was performed in five core Journals (The Lancet, JAMA, New England Journal of Medicine, British Medical Journal, Annals of Internal Medicine) and five medical education journals (Academic Medicine, Medical Education, BMC Medical Education, Medical Teacher, Teaching and Learning in Medicine and Education for Health) from April 1 until October 2009.

### Terms used

The Mesh terms (Medline Ovid)/Emtree terms (Embase) used were "Education, Medical, Graduate or Education" OR "Internship and Residency" OR "Family Practice/ed [Education]" as well as a combinations of terms relating to the WMFE framework items (quality, standards, legislation, education, organization, "organization and administration", *annual reports as topic, "constitution and bylaws", governing board, management audit, management information systems, mandatory programs, organizational innovation, program development, public health administration, total quality management).

In the ERIC database, the search was performed using the Thesaurus descriptors: "Graduate Medical Education" OR "Family Practice" AND keywords quality OR train* OR staff* OR standards OR organization OR legislation.

In the RDRB database, the search was performed using the key words: graduate medical education OR internship OR family practice combined with the key words: quality OR train* OR staff* OR standards OR organization OR legislation (Table 1).

**Table 1:** search strategy (Medline Ovid, Embase, ERIC, RDRB)

*1.1)MEDLINE*	*OVID*	
Keywords	3 MESHS	
	Education, Medical, Graduate/or Education/	
	"Internship and Residency"/	
	Family Practice/ed [Education]	
	AND	
	2 Free terms: quality - standards	
Date	April 23, 2009	
Database	Medline Ovid	
Search Strategy	1 exp Education, Medical, Graduate/or Education/	31768
	2 exp "Internship and Residency"/	26607
	3 Family Practice/ed [Education]	9044
	4 1 or 3 or 2	59921
	5 quality.mp.	412313
	6 standard.mp.	350030
	7 6 or 5	735413
	8 train*.mp.	231585
	9 8 and 4 and 7	2498
	10 limit 9 to ("review articles" and humans and (dutch or english or french) and last 14 years)	131
	11 from 10 keep 1-131	131
	12 4 and 7	5248
	13 limit 12 to ("review articles" and humans and (dutch or english or french) and last 14 years)	216
**Note**	**3 MESHs exploded combined with 2 free terms of interest (quality - standards)**	
Date	April 23, 2009	
Database	Medline OVID	
	17 Internship and Residency"/lj, ed, st, og [Legislation & Jurisprudence, Education, Standards, Organization & Administration]	4329
	18 Education, Medical, Graduate/lj, ed, st, og [Legislation & Jurisprudence, Education, Standards, Organization & Administration]	3329
	19 Family Practice/ed [Education]	9044
	20 Family Practice/og, lj, st [Organization & Administration, Legislation & Jurisprudence, Standards]	8460
	21 19 and 20	953
	22 21 or 18 or 17	7833
	23 limit 22 to ("review articles" and humans and yr = "1995-Current" and (dutch or english or french))	338
**Note**	**3 MESHs focused on the topics of interest: legislation - education - standards - organization**	
	**Merge databases: 338 + 216 = 554 papers - 94 duplicates**	
	**= 460 papers**	
Date	April 24, 2009	
Database	Medline Ovid	
Search Strategy	1 Exp Education, Medical, Graduate/or Education/	31769
	2 exp "Internship and Residency"/	26608
	3 Family Practice/ed [Education]	9044
	4 1 or 3 or 2	59923
	21 train*.mp.	231625
	33 staff*.mp.	140884
	34 *"organization and administration"/or *annual reports as topic/or *"constitution and bylaws"/or governing board/or management audit/or management information systems/or mandatory programs/or organizational innovation/or program development/or public health administration/or total quality management/	67323
	35 33 or 34 or 21	405544
	36 35 and 4	23424
	37 limit 36 to ("review articles" and humans and yr = "1995-Current" and (dutch or english or french))	646
**Note**	3 MESHs exploded combined with free terms (train* - staff*) and MESHs to reflect the WFME grid	
	**Merge databases: 460 + 646 = 1106 papers - 316 duplicates**	
	**= 790 papers**	
***1.2)EMBASE***		
Keywords	3 Emtree terms	
	"MEDICAL EDUCATION"/	
	"EDUCATION"/	
	"FAMILY PRACTICE"	
	AND	
	2 Free terms: quality - standards	
Date	June 9, 2009	
Database	Embase	
	1 'medical education'/exp AND [embase]/lim	99195
	2 'education'/exp AND [embase]/lim	302046
	3 'general practice'/exp AND [embase]/lim	25131
	4 #1 OR #3 OR #2	320689
	5 quality AND [embase]/lim	403812
	6 standard AND [embase]/lim	329464
	7 #5 OR #6	700932
	8 train* AND [embase]/lim	206593
	9 # 8 AND #4 AND #7	10658
	10 #4 AND #7	46774
	11 #4 AND #7 AND [review]/lim AND ([dutch]/lim OR [english]/lim OR [french]/lim) AND [embase]/lim AND [1995-2009]/py	7024
	12 #4 AND #7 AND ([meta analysis]/lim OR [randomized controlled trial]/lim OR [systematic review]/lim) AND [review]/lim AND ([dutch]/lim OR [english]/lim OR [french]/lim) AND [embase]/lim AND [humans]lim AND [embase]/lim AND [1995-2009]/py	392
**Note**	**3 Emtree terms combined with 2 free terms of interest (quality - standards)**	
Date		
June 9, 2009		
Database	Embase	
	13 legislation AND [embase]/lim	17350
	14 education AND [embase]/lim	315073
	15 standards AND [embase]/lim	54455
	16 organization AND [embase]/lim	243462
	17 #1 AND #13 AND #14 AND #15 AND #16	4
	18 #2 AND #13 AND #14 AND #15 AND #16	18
	19 #3 AND #13 AND #14 AND #15 AND #16	0
	20 #1 AND #13 AND #14 AND #15 AND #16 AND ([metaanalysis]/lim OR [randomized controlled trial]/lim OR[systematic review]/lim) AND [review]/lim AND ([dutch]/lim OR [english]/lim OR [french]/lim) AND [humans]/lim AND [embase]/lim AND [1995-2009]/py	0
	21 #13 OR #14 OR #15 OR #16 OR #19 OR #20	588503
	22 #1 AND #21	92453
	23 #13 OR #15 OR #16	307648
	24 #3 AND #23	2303
	25 #1 AND #23	10901
	26 #2 AND #23	36791
	27 #3 AND #24	2303
	28 #25 OR #26 OR #27	38427
	29 #25 OR #26 OR #27 AND ([meta analysis]/lim OR [ran	95
	domized controlled trial]/lim OR [systematic review/lim)	
	AND [review]/lim AND ([dutch]/lim OR [english]/lim	
	OR [french]/lim) AND [humans]/lim AND [embase]/lim	
	AND [1995-2009]/py	
**Note**	**3 Emtree terms focused on topics of interest: legislation - education - standards - organization**	
Date	June 9, 2009	
Database	Embase	
Search Strategy	1 'medical education'/exp AND [embase]/lim	99195
	2 'education'/exp AND [embase]/lim	302046
	3 'general practice'/exp AND [embase]/lim	25131
	5 #1 OR #3 OR #2	320689
	6 train* AND [embase]/lim	206655
	7 staff* AND [embase]/lim	73410
	8 'organization and management'/exp AND [embase]/lim	333505
	9 'health care management'/exp AND [embase]/lim	297404
	10 governing AND board AND [embase]/lim	214
	11 management AND audit AND [embase]/lim	5795
	12 management AND information AND systems AND	7583
	[embase]/lim	
	13 mandatory AND programs AND [embase]/lim	626
	14 organizational AND innovation AND [embase]/lim	240
	15 program AND development AND [embase]/lim	44213
	16 public AND health AND administration AND [embase]/lim	14407
	17 total AND quality AND management AND [embase]/lim	11402
	18 #7 OR #8 OR #9 OR #10 OR #11 OR #12 OR #13 OR #14 OR #15 OR #16	482585
	18 #5 OR #6 OR #17	700724
	19 #4 OR #18	113868
	20 #4 AND #18 AND ([meta analysis]/lim OR [randomized controlled trial]/lim OR [systematic review]/lim) AND [review]/lim AND ([dutch]/lim OR [english]/lim OR [french]/lim) AND [humans]/lim AND [embase]/lim AND [1995-2009]/p	391
**Note**	3 Emtree terms combined with free terms (train* - staff*) and MESHs to reflect the WFME grid	
	**Merge databases: 392 + 95 + 391 = 878 papers - 263 duplicates = 615 papers**	
***1.3) ERIC***		
Keywords	"Graduate Medical Education"	
	"Family Practice (medicine)"	
	quality	
	train*	
	staff*	
	standards	
	organization	
	legislation	
Date	July 30, 2009	
Database	ERIC	
Search Strategy	1) Thesaurus descriptors: "Graduate Medical Education"	
	OR	
	2) Thesaurus descriptors: "Family Practice (Medicine)	
	AND	
	3) Keywords: quality OR train* OR staff* OR standards OR organization OR legislation	
	Publication date: 1995-2010	
	Publication type: journal article	
	N = 303	
***1.4) RDRB***		
Keywords	graduate medical education	
	internship	
	residenc*	
	family practice	
	quality	
	train*	
	staff*	
	standards	
	organization	
	legislation	
Date	August 1, 2009	
Database	RDRB	
Search Strategy	Advanced search	
	X Search all groups	
	Key words: graduate medical education OR internship OR family practice	
	AND	
	Key words: quality OR train* OR staff*	
	Limits: 1995-2010	
	N = 255	
Date	August 1, 2009	
Database	RDRB	
Search Strategy	Advanced search	
	X Search all groups	
	Key words: graduate medical education OR residenc* OR family practice	
	AND	
	Key words: quality OR train* OR staff*	
	Limits: 1995-2010	
	N = 234	
	**Merge databases: 255 + 234 = 489 - 234 duplicates**	
	**= 255 papers**	
Date	August 1, 2009	
Database	RDRB	
Search Strategy	Advanced search	
	X Search all groups	
	Key words: graduate medical education OR internship OR family practice	
	AND	
	Key words: standards OR organization OR legislation	
	Limits: 1995-2010	
	N = 293	
	**Merge databases: 255 + 293 = 548 - 89 duplicates**	
	**= 459 papers**	

### Selection procedure

A total of 3022 unique references were identified. A first selection of reviews was performed by two independent researchers (in combinations AD, RR, JW) based on title and abstract using the following *inclusion *criteria:

• scope: quality of training programs, training practices, trainers;

• description of national, regional or official post graduate programs;

• study design: systematic reviews

**Figure 2:**
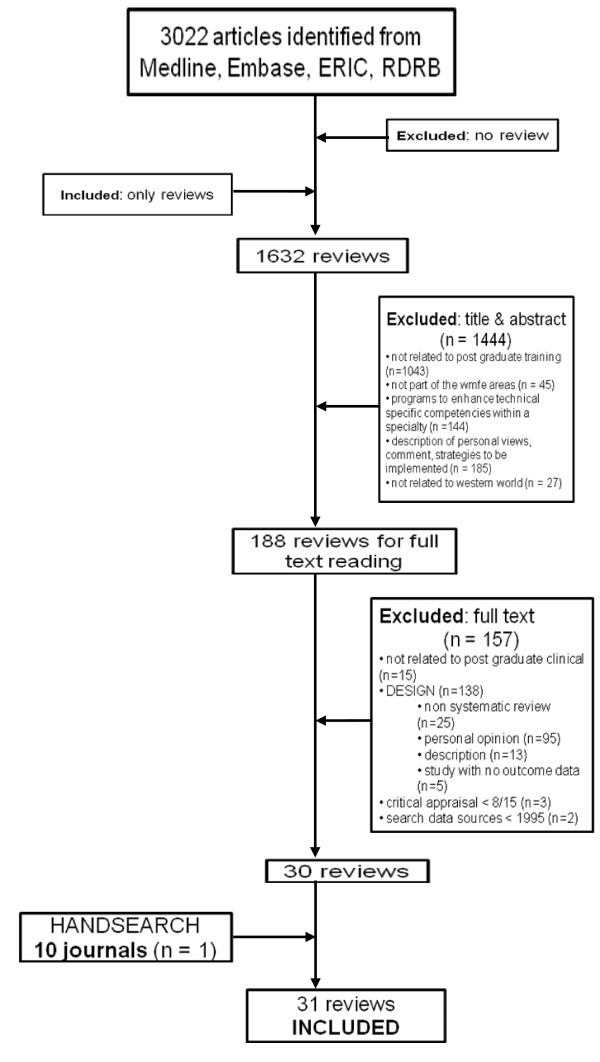
**Flow chart with selection process of reviews**.

Papers were excluded using the *exclusion *criteria mentioned in Figure 2. The percentage of agreement between assessors was 95.1 percent.

Disagreement was resolved in discussion by pairs of researchers. No arbitrating intervention by a third researcher was needed. Finally, 188 reviews were selected for full reading and quality appraisal. One systematic review found during the manual search completed the list.

### Quality appraisal

The quality appraisal of the selected reviews was performed on the full texts by one author (RR or JW) and was checked by the first author (AD). The researchers used the frameworks of Scottish Intercollegiate Network Group (SIGN) for reviews [12]. The maximal quality score was 15. The researchers excluded 3 reviews with low scores equal to 0 or 1 on three or more items out of a total of 5 items [13-15]. One review was excluded because it used, among other studies in the core curriculum, only one study in PME [16].

## Results

After the selection procedure, 31 reviews were selected for further analysis. Figure 2 overviews the selection process. Overall, the quality of the 31 reviews (assessed using the SIGN criteria) was high (≥ 12/15) in 23 reviews and moderate (8/15-12/15) in 8 reviews. The selected reviews [17-47] evaluated 1570 primary studies carried out in the context of PME. The reviews publication dates range from 2000 to 2009, of which 9 reviews were from January 2008 until October 2009.

Many reviews did not focus solely on PME. They also analysed primary studies of undergraduate training or even the education of other health professionals, for instance high-fidelity training in undergraduate medical education and continuing medical education [18].

Most training settings were not specified (n = 12) [19-22,27,29,31,33,35,37,45,46]. Some studies mentioned them i.e. hospital settings (n = 7) [17,18,34,41-44] and outpatient settings (n = 2) [24,47]. Ten papers studied a mixed setting [23,25,26,28,30,32,36,38-40] (i.e. hospital setting and/or GP setting and/or outpatient setting and/or not specified).

Looking at the first author's affiliations, the large majority of studies came from the US (n = 19) [17,18,21,23,24,26,28-31,33,36,37,39,40,42-44,47] and the UK (n = 5) [18,26,31,44,45]. Two first authors resided in Canada [25,41], 2 in Australia [22,35], 1 in the Netherlands [38], 1 in Spain [20] and 1 in Bahrain [34]. All papers were written in *English*.

### Practical messages

Additional file 1 gives an overview of the selected reviews: the research questions, the number of studies included in the reviews, the WFME (sub) areas that are covered, the results and the critical appraisal scores. The following paragraphs summarise their findings and highlight interesting and practical avenues to enhance the quality of PME. The following paragraphs use the term "trainee" for generic areas of PME training. The term "resident" is more specifically used when this trainee works in a clinical setting.

#### WFME areas under study

Most papers studied one particular area of the WFME framework while three other ones covered more than one area and/or sub-area [21,24,26]. The majority of the reviews give detailed information about the training process (WFME area 2) [17-26], the assessment of trainees (WFME area 3) [21,24,27-33] and the trainees (WFME area 4) [26,34-42]. One review covered the 'governance and administration' (WFME area 8) [24]. No review focused on the mission and outcomes, the evaluation of the training process and the continuous renewal (respectively areas 1, 7 and 9 of the WFME framework).

#### Importance of a core curriculum

The first step for the quality of post graduate medical training is the necessity to describe an effective "core" in-patient curriculum. That was the conclusion of Di Francesco et al. [17] who analysed the effectiveness of the training in internal medicine. They found little data on the topic and concluded that few data exist to support the quality of this training.

#### Conditions to facilitate learning in PME education: combination of learning approaches

Issenberg et al. found that high-fidelity simulations are educationally effective: they concluded that simulation based exercises could complement medical education in patient care settings [18]. High fidelity simulations use realistic materials and equipment to represent the task that the candidate has to perform. The authors also insist on four other conditions to facilitate learning i.e. feedback, repetitive practice, opportunities for the trainee to engage in the practice of medical skills across a wide range of difficulties and multiple learning strategies.

In the same way another review insists on the feedback to trainees as a key to success. It should be provided systematically over several years by an authoritative source. Feedback can change clinical performance, but its effects are influenced by the source and duration of the process [33].

#### Work in team: importance of the clinical settings

Working as a doctor implies working in teams. One systematic review has listed some principles (cfr additional file 1) to enhance the quality of teamwork among future specialists [23].

Furthermore, it is worthwhile to offer to the future hospital-based specialists adequate exposure to outpatient and ambulatory settings as an adjunct to training in inpatient settings [24]. This exposure leads to better performances on national Board examinations, Objective Structured Clinical Examinations and tests of clinical reasoning. Another review estimates that residents often lack of confidence and competence for common health issues because only 13% of the training takes place in ambulatory care [47].

#### Growing importance of the portfolio in post graduate medical training

A portfolio is a set of materials collected to represent a person's work and foster reflective learning. The use of a portfolio gains importance in PME training and authors also recommend it as a tool for assessment [21].

#### Selection and assessment of trainees: shortcomings of the academic results

The selection of trainees for PME positions remains a subjective exercise: the undergraduate grades and rankings moderately correlate with the performance during internship and residency [34]. A selection of trainees based only on previous academic results in the undergraduate curriculum poorly predicts the post graduate training.

Authors from an Australian study propose a mix of traits that could predict the success of a future candidate [35] i.e. communication skills, capacity of adaptation to the audience, empathy, understanding of the role of the nursing and supporting staffs, understanding of practice protocol.

#### Assessment of trainees: a call for a global approach

Most authors emphasize the need for a global assessment of the post graduate trainee. Many tools exist e.g. from the Objective Structured Clinical Examination [24], the Mini-CEX (Mini Clinical Evaluation Exercise = method of evaluating residents by directly observing a history and physical examination followed by feedback) [30]. In the US, the Accreditation Council for Graduate Medical Education considers the portfolio as a corner stone to evaluate the competences. A portfolio must have a creative component that is learner driven [21].

Epstein et al. emphasizes the need for a multidimensional assessment based on the observation of trainees in real situations, on feedbacks of peers and patients and on measures of outcomes. The assessment has to target many competencies e.g. professionalism, time management, learning strategies, teamwork [29]. The strong validity of evidence has been identified for the Mini-CEX but the authors conclude that more work is needed to review the optimal mix and the psychometric characteristics of assessment formats in terms of validity and reliability [24,30].

#### Working hours and risk of burnout: no optimal answer

Working conditions of trainees are a matter of debate in the literature. Work hours restrictions may improve the quality of life of the trainees, but it is unclear if the improved quality of life of residents ultimately results in better patient care [26]. Some evidence shows that reducing working hours does not impair clinical training, but it may decrease overall continuity of care [48,49]. It must be noted that American (80 hour work in the ACGME framework) and European directives (a maximum of 48 hours per week since August 2009 [49]) greatly differ.

Emotional exhaustion and burnout rates in medical residents are high: authors suggest a prevalence ranging from 18% to 82% in medical residents [38]. The personal and professional consequences are potentially dramatic [39,40] but few interventions are set up to tackle the problem. Support groups and meditation-type practices have shown promising results but are sometimes hard to replicate [40]. Doctor-nurse substitution has the potential to reduce doctors workload and direct health costs. Trainees welcome these reforms but trainers show reservations [50].

## Discussion

### Some evidence in reviews to build a post graduate training process

The reviews included in this review show that evidence in the reviews is available for training processes, assessment of trainees, and trainee processes. Course organisers, colleges and the stakeholders involved in quality of post graduate medical education should rely on this available evidence to build quality frameworks and assessments. The review suggests other pertinent criteria than the undergraduate curriculum to select the candidates for post graduate medical education. There is a need to define a core curriculum and to combine learning strategies, including simulations and high quality feedback from an authoritative source. The choice of clinical settings is crucial, in particular the opportunity to work in team and to care for common ailments seen in ambulatory practice. The papers finally provide exhaustive reviews of the available assessment instruments: however they underline the need for a better assessment of their validity and for an evolution towards a multidimensional assessment.

Some gaps in the literature have been identified e.g. on sub-areas of staffing, training settings, evaluation of training process. As an illustration the link between staff performance and training outcome and the relationship between training and service are hardly addressed in reviews. The selected reviews focus on one or several parts of the WFME framework but do not take into account their interactions.

### Strengths of this systematic review

This systematic review was comprehensive, based on the most important indexed databases for medical education topics. The selection of papers relied on strict criteria and the quality of the included papers was further assessed [12]. The authors who performed this review came therefore across all challenges mentioned by Reed and colleagues for performing systematic reviews of educational interventions, i.e., finding reports of educational interventions, assessing quality of study designs, assessing the scope of interventions, assessing the evaluation of interventions, and synthesizing the results of educational interventions [9].

### Limitations of this systematic review

Some limitations inherent to the methodology of this systematic review need to be addressed. An important limitation is linked to the choice of key words and search strings. The concept of quality covers a broad spectrum: the choice and combination of similar MESH and non MESH terms was difficult across all databases.

A second limitation relates to the authors' decision to focus on indexed literature only. Some publications from the grey literature have been probably missed but there is a risk to that these lack scientific rigor [1]. Another source of incomplete data source might be the decision to exclude information from reviews that focused on one specialty or one technical procedure in medicine only.

A third limitation is the questionable quality of the primary studies as reported by many authors of the systematic reviews. They noted that most primary studies relate to single institutions and that the designs of included studies were often of poor quality. (Randomised) controlled trials were seldomly included in the reviews. This means that currently the best evidence in the reviews on quality in PME relies on descriptive and cohort studies and before-and-after measurements.

The classification of papers within the appropriate areas and sub-areas was a challenge. Some misclassifications might arise from the subjective assessment of the researchers' team. In particular, some papers apply to more than one (sub) area. For instance, the paper of Carraccio [21] stressed the applicability of portfolios in assessment of trainees (area 3.1), but because of the formative applications it could also be placed in training content (area 2.3), as it represents what trainees do and reflects upon the content of their work. For reasons of clarity, these three reviews were therefore reported in two most relevant (sub) areas.

The mixed populations in the studies is finally also a possible limitation to the interpretation of the results. Often other groups than residents were included in the reviews (i.e. medical students, nurses) which makes it hard to identify evidence specific for the group of post graduate trainees. Educational needs of senior trainees are different from these of junior ones. The heterogeneity within reviews is of concern and should be identified more precisely by future reviewers.

## Conclusions

This systematic literature review identified and analyzed the available evidence for some areas of the post graduate training. The majority of the reviews provided information about the training process, the assessment of trainees and the trainees. Indicators used for quality assessment purposes of post graduate training should be based on this evidence but further research is needed for some areas in particular to assess the quality of the training process.

### Key Points of this systematic literature review

• This systematic review identified the available good quality reviews for some areas of the WFME framework: training approaches, assessment of trainees and working conditions are the areas most often reviewed;

• Useful criteria to select the candidates for post graduate medical education exist;

• There is a need to combine learning strategies: high quality feedback from an authoritative source is a key of success;

• A special attention is required for the choice of clinical settings: experiences in teams and practice in ambulatory care are essential to develop the trainee's competences;

• Many assessment tools are available but there is need for a better assessment of their validity and for an evolution towards a multidimensional assessment;

• This review identified a gap in reviewed research for some (sub) areas of the WFME framework: mission and outcomes, the evaluation of the training process and the continuous renewal.

## Competing interests

The authors declare that they have no competing interests.

## Authors' contributions

AD, RR and DP designed this study. AD, RR and JW selected and analysed data. AD, RR and JW drafted the manuscript. All authors read and approved the final version of the manuscript.

## Pre-publication history

The pre-publication history for this paper can be accessed here:

http://www.biomedcentral.com/1472-6920/11/80/prepub

## Supplementary Material

Additional file 1 **Summary of general findings of the selected 31 reviews**. Click here for file
