# Supplementary material for: Evidence based post graduate training. A systematic review of reviews based on the WFME quality framework
Source: BMC Med Educ. 2011 Oct 6;11:80. doi: 10.1186/1472-6920-11-80 (PMC3200166; doi:10.1186/1472-6920-11-80)
Supplement: Additional file 1 — Summary of general findings of the selected 31 reviews. [file 1472-6920-11-80-S1.DOC]

| **WFME AREA** | **WFME SUB-AREA** | **NUMBER OF STUDIES INCLUD-ED** | **RESEARCH**  **QUESTION** | **AUTHOR’S CONCLUSIONS** | **SIGN score** | **REF** |
| --- | --- | --- | --- | --- | --- | --- |
| 1. MISSION AND OUTCOMES | 1.1 STATEMENTS OF MISSION AND OUTCOMES |  |  | No review |  |  |
|  | 1.2 PARTICIPATION IN THE FORMULATION OF MISSION AND OUTCOMES |  |  | No review |  |  |
|  | 1.3 PROFESSIONALISM AND AUTONOMY |  |  | No review |  |  |
|  | 1.4 TRAINING AND OUTCOMES |  |  | No review |  |  |
| 2. TRAINING APPROACHES | 2.1 LEARNING APPROACHES | 13 | To review the available literature on specific internal medicine inpatient educational interventions and proposes recommendations for improving internal medicine training in this setting. | There is need to formally define and study what constitutes an effective “core” inpatient curriculum in internal medicine training. Few data exist on the effectiveness of the training of internal medicine curriculum in inpatient settings. New inpatient team structures and skills will be required to enhance the link and training in both the inpatient and outpatient settings. | 12/15 | DiFrances-co, 2005. [17] |
|  |  | 109 | What are the features and uses of high-fidelity medical stimulations that lead to effective learning? | High fidelity simulations are educationally effective and simulation-based education complements medical education in patient care settings. The top five of right conditions facilitating learning are:  * Providing feedback;  * Repetitive practice;  * Integration simulation-based exercises into the standard medical school or post graduate educational curriculum is an essential feature of their effective use;  * Effective learning is enhanced when learners have opportunities to engage in practice of medical skills across a wide range of difficulty level;  * Multiple learning strategies. | 14/15 | Issenberg, 2005. [18] |
|  | 2.2 SCIENTIFIC METHODS | 46 | What is the evidence that postgraduate teaching in evidence based medicine changes anything? | Teaching ´Evidence Based Practice´ gives a small improvement in knowledge, skills, attitudes or behaviour. The workshop was the most frequent intervention followed by the multifaceted intervention. | 15/15 | Coomarasamy, 2004.[19] |
|  |  | 24 | To systematically review studies that assessed the effectiveness of EBP (Evidence-based-practice) teaching to improve knowledge, skills, attitudes and behavior of postgraduate healthcare workers, and to describe instruments available to evaluate EBP teaching. | Small improvements in knowledge, skills, attitudes or behavior are noted when measured alone. A large improvement in skills and knowledge in EBP is noted when measured together in a total score. Very few studies used validated measures tests. | 15/15 | Flores-Mateo, 2007. [20] |
|  | 2.3 TRAINING CONTENT | 30 | Evaluating competence using a portfolio: a literature review and web-based application to the ACGME competencies | A portfolio is a set of materials collected to represent a person's work. The use of a portfolio allows to incorporate a variety of tools in order to foster reflective learning, which is the key to professional development. Web-based portfolio assessment provides a venue for the evaluation of competence and has the ability to provide educators with a research infrastructure to practice evidence-based education. | 10/15 | Carraccio, 2004. [21] |
|  |  | 44 | To evaluate the effectiveness of medical skills laboratories or simulators. Is performance in medical skills laboratories transferable to actual clinical performance and maintained over time? | Medical skills laboratories do lead to improvement in procedural skills compared with standard or no training at all when assessed by simulator performance and immediately post training.  The most common procedural skills taught in medical laboratories were laparoscopic surgery skills (54%), followed by other surgical skills (including endoscopy, endo-urological skills, urological skills, bronchoscopy and other general surgical skills (30%). The remaining 16% evaluated the effectiveness of simulators in training in resuscitation, catheterization, trauma management, anaesthesia, and cardiac life support skills. | 14/15 | Lynagh, 2007. [22] |
|  |  | 13 | To assess the characteristics and efficacy of published curricula designed to teach teamwork to medical students and house staff. | Some curricula address teamwork principles. The eight following teamwork principles were identified:  * Leadership training or elevated leadership skills;  * Team members monitor one another’s performance and provide feedback (mutual performance monitoring);  * Redistributing tasks upon demand by anticipating team member’s needs through accurate knowledge of their responsibilities (backup behavior);  * The ability to adapt to changing situations;  * Soliciting team member ideas in defining goals and objectives (team orientation);  * Fostering trust between team members;  * Including communication training or evaluated communication skills.  * The curriculum addressed ensuring that team members are “on the same page” (shared mental models). | 15/15 | Chakrabor-ti, 2008.[23] |
|  | 2.4 TRAINING STRUCTURE COMPOSITION AND DURATION | 140 | 1. Are students and residents learning in ambulatory settings? 2. What characterizes an optimal ambulatory learning environment? 3. Are teachers and learners satisfied with their ambulatory care learning experiences?  4. What are the costs of teaching in these settings? | The ambulatory environment might act as an adjunct to inpatient training, as demonstrated by their performances on national board examinations, OSCEs, and tests of clinical reasoning. | 10/15 | Bowen, 2002.[24] |
|  |  | 74 | To review empirical evidence and theory pertaining to the role of progressive autonomy in clinical learning | Clinical trainees act more independently as their training progresses. However, no studies were identified that showed the educational efficacy of providing progressive independence, or the consequences of failing to do so. | 15/15 | Kennedy, 2005.[25] |
|  | 2.5 THE RELATIONSHIP BETWEEN TRAINING AND SERVICE |  |  | No review |  |  |
|  | 2.6 MANAGEMENT OF TRAINING | See 2.1 | See 2.1 | There is a pressing need to reconsider the current structure of inpatient education in internal medicine. New approaches to supervision, teaching and evaluation will be required. Authors concluded that the core should consist of most common medical conditions, a core set of necessary inpatient procedures and the system based practice skills relevant to inpatient setting. | 12/15 | Di Francesco, 2005. [17] |
|  |  | 54 | To summarize the literature regarding the effect of interventions to reduce resident work hours on residents' education and quality of life. | Interventions to reduce work hours (night and day float teams, extra cross coverage and physician extenders) resulted in mixed effects on both operative experience and on a perceived educational quality. Potential unintended consequences of reducing resident work hours included inadequate development of professionalism, worse patient – physician communication and a decrease in experience. | 15/15 | Fletcher, 2005. [26] |
| 3. ASSESSMENT OF TRAINEES | 3.1 ASSESSMENT METHODS | 75 | 1. Is it possible to identify generic criteria which would encapsulate the criteria in existing checklists? 2. To quantify the extent to which existing checklists allow assessment of humanistic and team competencies (i.e. to examine the validity of current checklists for holistic assessment of clinical procedural skills). | For clinical procedural skills, it is possible to develop generic criteria for the global assessment in professional groups (doctors, residents). A clinical procedure is a social interaction and a competent practitioner will perform the procedure correctly in a patient-centered way in the broader context of healthcare and healthcare team”. Seven main themes for clinical procedural skills were identified:  * Procedural competence;  * Preparation;  * Safety;  * Communication and working with the patient;  * Infection control;  * Post procedural care;  * Team working | 12/15 | McKinley, 2008. [27] |
|  |  | See 2.3 | See 2.3 | Portfolios can next to assist reflective learning and assisting in the training content, also be used for assessment purposes. It allows one to incorporate a variety of assessment tools needed to evaluate the broader ACGME domains of competence. Furthermore, the portfolio aids reflective learning, which is the key to professional development. | 10/15 | Carraccio, 2004. [21] |
|  |  | 56 | To evaluate published evidence that the ACGME's six competencies can each be measured in a valid and reliable way.  1. PATIENT CARE 2. MEDICAL KNOWLEDGE 3. PRACTICE BASED LEARNING AND IMPROVEMENT 4. INTERPERSONAL AND COMMUNICATION SKILLS 5. PROFESSIONALISM 6. SYSTEM-BASED PRACTICE | It is recommend using the ACGME competencies to guide and coordinate specific evaluation efforts, rather than attempting to develop instruments to measure the competencies directly. Peer-reviewed literature provides no evidence that current measurement tools can assess the competencies independently of one another. | 15/15 | Lurie, 2009.[28] |
|  |  | 195 | To propose a definition of professional competence, to review current means for assessing it, and to suggest new approaches to assessment. | The best assessment would be to observe trainees in real – life situations, incorporate the perspectives of peers and patients, or use measures that predict clinical outcomes. The literature on such assessments is scarce. In addition to assessments of basic skills, new formats that assess clinical reasoning, expert judgment, management of ambiguity, professionalism, time management, learning strategies, and teamwork require a multidimensional assessment while maintaining adequate reliability an validity. | 13/15 | Epstein, 2002. [29] |
|  |  | See 2.4 | See 2.4 | Numerous assessment methods are identified, among which standardized OSCE, the Mini Clinical Evaluation Exercise (Mini-CEX = method of evaluating residents by directly observing a history and physical examination followed by feedback). Between 12 and 14 encounters are required to obtain a reproducibility score of .80. | 10/15 | Bowen, 2002. [24] |
|  |  | 85 | To identify observation tools used to assess medical trainees' clinical skills with actual patients and to summarize the evidence of their validity and outcomes. | Although many tools are available for the direct observation of clinical skills, validity evidence and description of educational outcomes are scarce. The strongest validity evidence has been established for the Mini-CEX.[31] | 9/15 | Kogan, 2009.[30] |
|  |  | 191 | 1) to extend and update the work of previous authors concerning assessing professionalism 2) different from the format of previous reviews, to provide a catalog of the many and varied assessments available in the literature | Useful approaches to assessing resident physician professionalism include a 360-degree assessment and a cognitive assessment. Instead of creating new professionalism assessments, existing assessments should be improved. Also, more studies on the predictive validity of assessments and their use as part of formative evaluation system are recommended. | 13/15 | Lynch, 2004. [31] |
|  |  | 55 | To collate the published works on validation on assessments used in postgraduate medical certification. | There is a relative scarcity of published papers on validation of assessment for post graduate medical certification. The papers in this study demonstrate that good practice in test development and implementation is present in medicine but there is insufficient evidence to support the validity and reliability of any single assessment process. | 14/15 | Hutchinson, 2002. [32] |
|  | 3.2 RELATION BETWEEN ASSESSMENT AND TRAINING |  |  | No review |  |  |
|  | 3.3 FEEDBACK TO TRAINEES | 41 | To summarize evidence related to the impact of assessment and feedback on physicians' clinical performance. | Feedback can change physicians' clinical performance when provided systematically over multiple years by an authoritative and credible source.  The effects of formal assessment and feedback on physician performance are influenced by the source and duration of feedback. Other factors, such as physicians' active involvement in the process, the amount of information reported, the timing and amount of feedback, and other concurrent interventions, such as education, guidelines, reminder systems and incentives, also appear to be important. However, the independent contributions of these interventions have not been well documented in controlled studies. Conclusions are very limited, as most studies for this subgroup were conducted for one year or less. | 15/15 | Veloski, 2006.[33] |
| 4. TRAINEES | 4.1 ADMISSION POLICY AND SELECTION | 38 | To what extent do measurements obtained in medical schools predict outcomes in clinical practice: performance during internship, residency programs, on the job and its impact on healthcare? | Undergraduate grades and rankings were only moderately correlated with internship and residency performance. Existing assessment tools like the National Board Medical Examinations (NBME) I, II and III do appear to have low to moderate correlation with post graduate training performance. Little is known about their relationship to longer term practice. | 15/15 | Hamdy, 2006. [34] |
|  |  | 18 | To ascertain the specialized communication issues clinicians need to understand when preparing international medical graduates (IMGs) for clinical practice in Australia. | Traits of foreign candidates that could predict successful future training are:  * The ability to communicate with a range of people;  * The ability to choose the appropriate terminology, register, and amount of information for different audiences;  * Empathy;  * The skills to interact with nursing staff, and a clear understanding of the role of support staff in clinical care;  * An understanding of practice protocols with ongoing monitoring of whether information is being interpreted accurately. | 13/15 | Pilotto, 2007. [35] |
|  | 4.2 NUMBER OF TRAINEES |  |  | No review |  |  |
|  | 4.3 SUPPORT AND COUNSELLING OF TRAINEES | 52 | To illuminate areas of the model and test its construction against literature on resident performance. | Few discrete associations between the individual resident’s factors (learning styles/ personality, social/financial factors, practice preferences, personal health, and response to job environment) and actual job performance were identified. Few studies in this review examined final performance outcomes. Most of the included studies were single institution, cross-sectional and survey-based studies. This review paints a distressing picture of physicians in training. Stress, burnout and fatigue levels are high. Levels of depression vary and can be quite high (7-15%). They also identified three studies where residents during their training had increased empathy. This review did not address possible solutions for these observations. | 12/15 | Mitchell, 2005.[36] |
|  |  | 59 | To review the literature, and provides perspectives and recommendations from other sources to comprehensively examine four vital issues related to the problem: identification, underlying causes, management and prevention | Problem residents represent a significant challenge to medical educators. The prevalence is between 4-15%. Factors that are thought to be supportive to house officers, and may even prevent various problems are:  * Early detection through timely evaluations;  * Prompt specific feedback and discussion of concerns;  * Orientation and communication of expectations at the beginning of every year;  * Advisor/advisee system;  * Faculty role models;  * Close resident camaraderie;  * Support group among residents;  * Planned social events, retreats;  * Promotion of self-awareness and self-care. | 10/15 | Yao, 2001. [37] |
|  | 4.4 WORKING CONDITIONS | 19 | 1. What do we know about the prevalence of burnout in medical residents? 2. What are the risk and resistance factors that contribute to or prevent burnout in medical residents? | This review pointed burnout rates between 18 to 82%. Predictors of burnout can be characterised as either occupational or individual. Four of the 16 occupational risk factors (i.e. quantitative work overload, increased perception of work as stressful, an increase in anticipation of debt at the end of training and increased conflict between work and home) appeared to be strongly related to burnout. | 15/15 | Prins, 2007. [38] |
|  |  | 15 | 1) What is the level of significant burnout among residents? 2) What factors are associated with development of burnout? 3) What are the health and performance consequences for residents with burnout and their patients? 4) What coping resources may help residents with burnout? | Young physicians who readily embraced hard work in premedical and undergraduate medical education experience high levels of professional burnout in residency training years. Aside from working long hours, something about residency seems to leave many residents feeling emotionally exhausted and cynical and leaves some depressed and critical of their own patient care performance as well. | 10/15 | Thomas, 2004. [39] |
|  |  | 9 | To review Resident Physicians` Burnout | Burnout is highly prevalent among resident physicians and medical students. Despite the potentially serious personal and professional consequences of burnout, few interventions exist to solve this problem. Prospective, controlled studies are needed to examine the effects of interventions to manage burnout among resident physicians. Only 2 studies were RCT’s and many studies used volunteers. The use of support groups and meditation-type practices was very flexible and hard to replicate, although showing some promising results. | 14/15 | McCray, 2008. [40] |
|  |  | 29 | The author reviews the issues surrounding pregnancy during residency by evaluating published commentaries | This review suggested an increased risk of complications, especially adverse late-pregnancy events. Pregnant residents found the physical demands of residency and lack of support from fellow residents and their departments most stressful. | 10/15 | Finch, 2003.[41] |
|  |  | See 2.6 | See 2.6 | Residents' quality of life may improve with work hour limitations, but interpretations of the outcomes of these studies is hampered by suboptimal study design and the use of non validated instruments. The long-term impact of reducing resident work hours on educational quality and patient outcomes remains unknown. So, it is unclear if the improved quality of life of residents ultimately results in better patient care. | 15/15 | Fletcher, 2005.[26] |
|  |  | 16 | To analyze resident physicians' activities to assess educational value of residents' work | It is potentially valuable to consider not only the number of hours worked by residents, but the educational content of their work when considering residency work and work hour reforms debate. To analyse resident physician activities for educational value, it showed that approximately 15% was directed to programs teaching activities, 36% of time was directed to patient care to specialty-specific learning objectives, and 35% to delivering patient care to marginal or no educational value. | 15/15 | Boex, 2003.[42] |
|  | 4.5 TRAINEE REPRESENTATION |  |  | No review |  |  |
| 5. STAFFING | 5.1 APPOINTMENT POLICY |  |  | No review |  |  |
|  | 5.2 OBLIGATIONS AND DEVELOPMENT OF TRAINERS |  |  | No review |  |  |
|  |  | 24  13 | To provide an updated systematic review of the literature on residents –as - teachers curricula to determine the most evidence – based curricula and evaluation strategy.  (1) identify all RCT's for residents' teaching skills (2)identify the efficacy of those interventions of improving skills (3) identify the strengths and weaknesses of the available studies across medical disciplines 4) identify currently available methods for enhancing residents' teaching skills for residents training in psychiatry. | We did not find any systematic review to fit strictly in the two sub-areas of this standard i.e., ‘appointment policy’ and ‘obligations and development of trainers’.  Two reviews went into the educational value of residents themselves, which may be important in the continuum, i.e the teaching of undergraduates by residents. These reviews concluded that resident-as-teachers curricula can significantly improve residents' teaching skills. Nevertheless, there is a paucity of evidence that training programs work, especially concerning psychiatry residents-as-teachers programs. | 13/15  15/15 | Post, 2009.[43]  Dewey, 2008.[44] |
| 6. TRAINING SETTINGS AND EDUCATIONAL RESOURCES | 6.1 CLINICAL SETTINGS ANS PATIENTS | 47 | To provide a summary of evidence for the role and effectiveness of real patient involvement in medical education | Patient involvement in medical education is becoming important. There were several examples of how to recruit and train patients to perform an educational role. The effectiveness of patient involvement was measured by evaluation studies and reported improvement in clinical skills and knowledge about the disease or condition or about social aspects of the disease. Although there is evidence of the short-term positive impact of patient involvement in medical education, evidence of longer-term impact is still lacking. Issues of ethics, psychological impact and influence on educational policy were poorly explored. | 15/15 | Jha, 2009.[45] |
|  | 6.2 PHYSICAL FACILITIES AND EQUIPMENT |  |  | No review |  |  |
|  | 6.3 CLINICAL TEAMS |  |  | No review |  |  |
|  | 6.4 INFORMATION TECHNOLOGY |  |  | No review |  |  |
|  | 6.5 RESEARCH |  |  | No review |  |  |
|  | 6.6 EDUCATIONAL EXPERTISE | 57 | To review the literature on effective supervision in practice settings in order to identify what is known about effective supervision. | There is no good quality research on effective supervision. Current supervisory practice in medicine has very little empirical or theoretical basis. Available data show there is some evidence that the supervision relationship is probably the single most important factor for the effectiveness of supervision, more important than the supervisory methods. Feedback is essential and must be clear so that the trainee learns his/her strengths and weaknesses. It is important that the trainee has some control over and input into the supervisory process. Finding sufficient time for supervision can be a problem. | 11/15 | Kilminster, 2000.[46] |
|  | 6.7 TRAINING IN OTHER SETTINGS AND ABROAD | 55 | To review the literature on ambulatory education and make recommendations for change. | No study assessed the overall effectiveness for ambulatory training in internal medicine. It showed that only about 13% of the time was spent in ambulatory care. Several studies consistently showed that residents lack confidence and competence for many common health issues. | 10/15 | Bowen, 2005.[47] |
| 7. EVALUATION OF TRAINING PROCESS | 7.1 MECHANISM FOR PROGRAMME EVALUATION |  |  | No review |  |  |
|  | 7.2 FEEDBACK FROM TRAINERS AND TRAINEES |  |  | No review |  |  |
|  | 7.3 USING TRAINEE PERFORMANCE |  |  | No review |  |  |
|  | 7.4 AUTHORISATION AND MONITORING OF TRAINING SETTINGS |  |  | No review |  |  |
|  | 7.5 INVOLVEMENT OF STAKEHOLDERS |  |  | No review |  |  |
| 8. GOVERNANCE AND ADMINISTRATION | 8.1 GOVERNANCE |  |  | No review |  |  |
|  | 8.2 PROFESSIONAL LEADERSHIP |  |  | No review |  |  |
|  | 8.3 FUNDING AND RESOURCE ALLOCATION | See 2.4 | See 2.4 | Offering education possibilities in medical centres costs efforts. Most of the studies in this review were single institution studies which limits the possibility to generalise the findings. Overall clinical teaching in ambulatory settings receive high ratings from students and residents. Clinical preceptors who accept students and residents in their practices do not tend to reduce their clinical loads, but they seem to extent their work days with 30-50 minutes per half day clinic. No data were reported for structural facilities (rooms, technical facilities etc.) Teaching settings in ambulatory care costs about one third more than non teaching settings to operate. | 10/15 | Bowen, 2002. [24] |
|  | 8.4 ADMINISTRATION |  |  | No review |  |  |
|  | 8.5 REQUIREMENTS AND REGULATIONS |  |  | No review |  |  |
| 9. CONTINUOUS RENEWAL |  |  |  | No review |  |  |
